# Fabrication of a Cell-Friendly Poly(dimethylsiloxane) Culture Surface via Polydopamine Coating

**DOI:** 10.3390/mi13071122

**Published:** 2022-07-15

**Authors:** Da Hyun Yang, Sangyong Jung, Jae Young Kim, Nae Yoon Lee

**Affiliations:** 1 Department of BioNano Technology, Gachon University, 1342 Seongnam-daero, Sujeong-gu, Seongnam-si 13120, Korea; jieuny94@gachon.ac.kr; 2 Institute of Molecular and Cell Biology (IMCB), Agency for Science, Technology and Research (A*STAR), Singapore 138667, Singapore; Jung_Sangyong@imcb.a-star.edu.sg or; 3 Department of Physiology, Yong Loo Lin School of Medicine, National University of Singapore, Singapore 117593, Singapore; 4 Department of Life Science, Gachon University, 1342 Seongnam-daero, Sujeong-gu, Seongnam-si 13120, Korea

**Keywords:** cell adhesion, poly(dimethylsiloxane), polydopamine, human umbilical vein endothelial cells, mesenchymal stem cells, vascular mimetics

## Abstract

In this study, we fabricated a poly(dimethylsiloxane) (PDMS) surface coated with polydopamine (PDA) to enhance cell adhesion. PDA is well known for improving surface adhesion on various surfaces due to the abundant reactions enabled by the phenyl, amine, and catechol groups contained within it. To confirm the successful surface coating with PDA, the water contact angle and X-ray photoelectron spectroscopy were analyzed. Human umbilical vein endothelial cells (HUVECs) and human-bone-marrow-derived mesenchymal stem cells (MSCs) were cultured on the PDA-coated PDMS surface to evaluate potential improvements in cell adhesion and proliferation. HUVECs were also cultured inside a cylindrical PDMS microchannel, which was constructed to mimic a human blood vessel, and their growth and performance were compared to those of cells grown inside a rectangular microchannel. This study provides a helpful perspective for building a platform that mimics in vivo environments in a more realistic manner.

## 1. Introduction

Over the past few decades, many researchers have attempted to effectively culture cells in vitro by mimicking in vivo microenvironments. In particular, microfluidic chips have been actively studied, as the dimensions of microfluidic channels and the growing conditions of cells in them are similar to those observed in human blood vessels [1]. For example, a microfluidic chip model mimicking the blood–brain barrier, which is a biological barrier located in the vasculature of the human brain, has been fabricated [2], and a microfluidic organ-on-chip system has been constructed for the real-time monitoring of cellular metabolites [3]. Furthermore, it has been shown that microfluidic platforms can enable the development of organ-on-a-chip devices to study cancer metastasis [4] as well as diagnostic models to test for diseases [5,6] and screen for drugs [7]. The advantage of using a fluidic chip lies in the dimensional similarity of the microfluidic channel to human microenvironments, particularly human vasculature.

Poly(dimethylsiloxane) (PDMS) has played a pivotal role as the primary material used to fabricate microfluidic chips. Many researchers have employed PDMS due to its cost-effectiveness; easy fabrication processing via replica molding; and high permeability to gases, which is very important for the continued supply of oxygen and removal of carbon dioxide, as well as for the stable maintenance of the culture medium’s pH [8,9]. Nevertheless, due to the inherent hydrophobicity and low surface energy of PDMS, it is difficult to successfully culture cells on its surface. Adhesion is the first stage of successful cell culturing after seeding, but the hydrophobic nature of PDMS generally prevents cells from adhering, therefore hindering cell proliferation. Consequently, many studies have been conducted aiming to modify PDMS into a cell-friendly surface, and it has been reported that various approaches improve cell adhesion and proliferation without causing fatal defects to cells [10,11,12,13,14]. Long et al. applied a polyethylene glycol (PEG)-coated layer to increase the hydrophilicity of the PDMS microfluidic chip [12], and Cho et al. coated PDMS with fibronectin, which is one of the extracellular matrix (ECM) proteins, to study the trophoblast invasion of human umbilical vein endothelial cells (HUVECs) [13]. In addition to these chemistry- and biology-based approaches, physics-based attempts to alter surface wettability, such as plasma-based treatment [14] and the application of corona discharge, have also been actively used to modify the surface hydrophobicity of PDMS [15]. Nonetheless, these modification methods have some drawbacks, such as the loss of cell stability, the recovery of the innate hydrophobic surface, or the necessity of the use of multi-step processes to coat PDMS surfaces.

Polydopamine (PDA) has emerged as a promising coating reagent for surface modification and is therefore considered one of the most appropriate substances for resolving issues associated with the use of PDMS. PDA is a protein-based adhesive of a naturally derived ingredient found in the foot of mussels [16]. PDA polymerization occurs under simple oxidative alkaline conditions [16] and effectively mediates the adhesion of cell growth factors and ECM proteins onto various substrates coated with PDA via Michael addition or Schiff base reaction [17,18,19,20]. In addition, PDA can be used to coat various surfaces, including metals, ceramics, and polymers [16,21]. Moreover, even if the surface of a material is originally hydrophobic, it can be converted to hydrophilic with long-term stability [17,18,19,21,22]. Thus, PDA is increasingly being studied and employed in cell research due to its universal good coating performance and ability to adhere to diverse cellular molecules. For instance, it has been reported that PDA-coated surfaces can reduce the oxidative stress caused to cells by reactive oxygen species [23] and have cytochemical advantages, such as the ability to differentiate cells [24], stabilize cell interaction [25], and retain a stable coating even within cells [26].

In this study, we coated a PDMS surface with PDA and measured the water contact angle to determine the optimum coating time to modify PDMS wettability. X-ray photoelectron spectroscopy (XPS) was then used to confirm the successful incorporation of chemical components into the PDMS surface. Human-bone-marrow-derived mesenchymal stem cells (MSCs) and HUVECs, which are different in nature, were cultured on the PDA-coated PDMS surface, and their growth and proliferation patterns were compared to those of cells grown on a bare PDMS surface. In addition, a cytotoxicity assay was performed to visualize the adhesion and proliferation of cells cultured on the PDA-coated surface and evaluate the viability of adherent cells. As MSCs are known to differentiate into either chondrocytes or osteoblasts, depending on the culture conditions, the effect of the PDA coating on MSC differentiation was also assessed. Moreover, a cylindrical microchannel mimicking human vasculature was fabricated, and HUVECs, endothelial cells lining the inner wall of blood vessels, were cultured inside the cylindrical microchannel, and their cell growth pattern, including morphology and proliferation, was compared with that of cells grown inside a rectangular microchannel. Overall, this study investigated the potential of using a PDA-coated PDMS surface as a platform to mimic human blood vessels in vivo.

## 2. Materials and Methods

### 2.1. Materials

Poly(dimethylsiloxane) (PDMS) pre-polymer (Sylgard 184) and a curing agent were purchased from Dow Corning (Midland, MI, USA). Tris-HCl was purchased from Biosesang (Seongnam, Korea). Dopamine hydrochloride and Alizarin Red S were obtained from Sigma-Aldrich (St. Louis, MO, USA). MSCs, HUVECs, MSC growth medium (MSCM), endothelial cell growth medium (ECM), endothelial cell growth supplements (ECGS), MSC growth supplements (MSCGS), fetal bovine serum (FBS), penicillin/streptomycin (P/S) solution, trypsin-EDTA (T/E) solution, Dulbecco’s phosphate-buffered saline (DPBS), MSC chondrogenic differentiation medium (MCDM), MSC chondrogenic differentiation supplement (MCDS), MSC osteogenic differentiation medium (MODM), and MSC osteogenic differentiation supplement A and B (MODS-A and MODS-B) were purchased from ScienCell (Carlsbad, CA, USA). Transforming growth factor-β3 (TGF-β3) and live/dead viability/cytotoxicity kits were purchased from Thermo Fisher Scientific (Waltham, MA, USA). Safranin O was obtained from Polyscience (Warrington, PA, USA). Norland optical adhesive63 (NOA63) was purchased from Norland Products (Jamesburg, NJ, USA). Polystyrene (PS) sheets and poly(ethylene terephthalate) (PET) film were obtained from Goodfellow (Pittsburgh, PA, USA).

### 2.2. PDMS Mold Fabrication and Surface Coating

**Figure 1:**
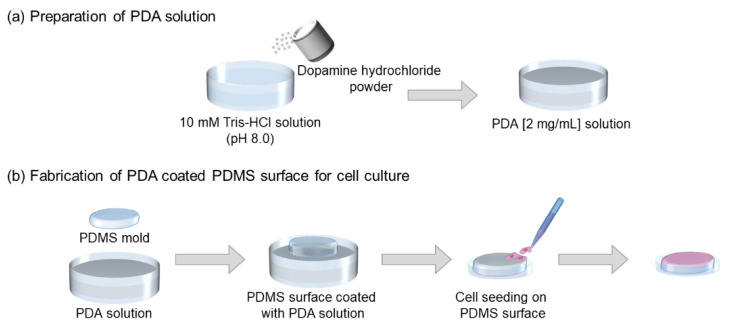
Schematic illustration showing the fabrication of a PDA-coated PDMS surface. (**a**) Preparation of the PDA solution and (**b**) application of PDA coating to the PDMS surface followed by cell seeding.

The PDMS pre-polymer was mixed with the curing agent at a 10:1 (*w*/*w*) ratio and degassed. The mixture was then baked at 80 °C for 90 min. To prepare the PDA solution, as shown in Figure 1, 1 M Tris-HCl buffer (pH 8.0) was diluted to 10 mM in deionized (DI) water, and dopamine hydrochloride powder was dissolved in the alkaline solution at a concentration of 2 mg/mL. To perform the coating, the PDMS mold was soaked in the PDA solution for 6 h; it was then rinsed twice with DI water and thoroughly dried at room temperature (RT). The PDA-coated PDMS surface was treated with 70% ethanol and sterilized for 90 min via UV irradiation; it was then placed in a six-well plate before being used as a cell culture surface.

### 2.3. Characterization of the PDMS Surface

#### 2.3.1. Water Contact Angle

To confirm that the PDMS surface had been converted to hydrophilic via the PDA coating process, the water contact angle was measured using a Phoenix 300 contact angle analyzer (Surface Electro Optics, Suwon-si, Gyeonggi-do, Korea) at the Core Facility for Bionano Materials, Gachon University, Korea. The parameter was calculated by averaging the value of water droplets placed in five different locations on the PDMS surface.

#### 2.3.2. X-ray Photoelectron Spectroscopy

X-ray photoelectron spectroscopy (XPS) analyses were performed to verify the successful PDA coating of the PDMS surface using an AxisHsi instrument (Kratos Analytical, Manchester, UK) equipped with an aluminum X-ray source (mono-gun, 1486.6 eV) with a pass energy of 40 eV. Before the data were recorded, the pressure in the chamber was less than 5 × 10^−9^ Torr, and the anode voltage and current were 13 kV and 18 mA, respectively. The take-off angle was set at 45° and the resolution for the binding energy measurement was approximately 0.1 eV. A binding energy of C1s (284.6 eV) was used as a reference.

### 2.4. Cell Culture on the PDA-Coated PDMS Surface

In all cell culture experiments on the PDA-coated PDMS surface, two types of cells were used from passage numbers 4 to 7. HUVECs were cultured in ECM containing ECGS, FBS, and penicillin/streptomycin. The cells were incubated in a 5% CO_2_ humidified incubator at 37 °C with an ECM that was changed every 2 to 3 days. When the cell confluency reached approximately 80%, the cells were trypsinized with trypsin/EDTA solution to obtain cell detachment and then collected. The collected cells were counted using a hemocytometer before being seeded on the six PDMS molds placed inside the six-well plate, one per well. The HUVECs were seeded at a density of 9.6 × 10^4^ cells per well. MSCs were cultured in MSCM with MSCGS, FBS, and penicillin/streptomycin solution. They were then incubated, harvested, and seeded under the same conditions used for HUVECs.

### 2.5. Live/Dead Cell Assay

HUVECs were cultured for 3 days to allow the cells to be attached and proliferated before a live/dead assay was carried out. The medium in the six-well plate was discarded and the plate was gently washed using DPBS. Calcein AM and ethidium homodimer-1 (EthD-1) were diluted in DPBS to 2 μM and 4 μM, respectively, and 1.5 mL of the solution was aliquoted into each well; the plate was then incubated at RT for 10 min before visualization using a fluorescence microscope. Live cells and dead cells were stained with calcein AM and EthD-1, respectively. A live/dead cell assay was carried out on MSCs using the same protocol, except for with a longer culture period of 7 days.

### 2.6. MSC Differentiation Staining

To allow the differentiation of MSCs into osteocytes and chondrocytes on the PDA-coated PDMS surface, cells were proliferated to 100% confluency [27]; MSCM was then replaced with MODM for osteogenesis and with MCDM for chondrogenesis. MODM supplemented with MODS-A, MODS-B, FBS, and P/S is known to promote osteogenesis, while MCDM containing MCDS, TGF-β3, and P/S induces chondrogenesis. During the 2-week period of differentiation, the differentiation medium was refreshed every 3 to 4 days. The cells were then fixed in 10% formalin for 1 h before being washed twice with DI water. The osteogenic cells differentiated on the PDA-coated PDMS were stained using 2% (*w*/*v*) Alizarin Red S solution for 45 min, while chondrogenic cells were stained using 0.1% (*w*/*v*) Safranin O solution for 15 min, both at RT. After staining, the plates were thoroughly rinsed several times with DI water to remove the remaining staining solution and photos were taken using an inverted optical microscope (IX71, Olympus).

### 2.7. Fabrication of the Cylindrical PDMS Microchannel for Cell Culture

**Figure 2:**
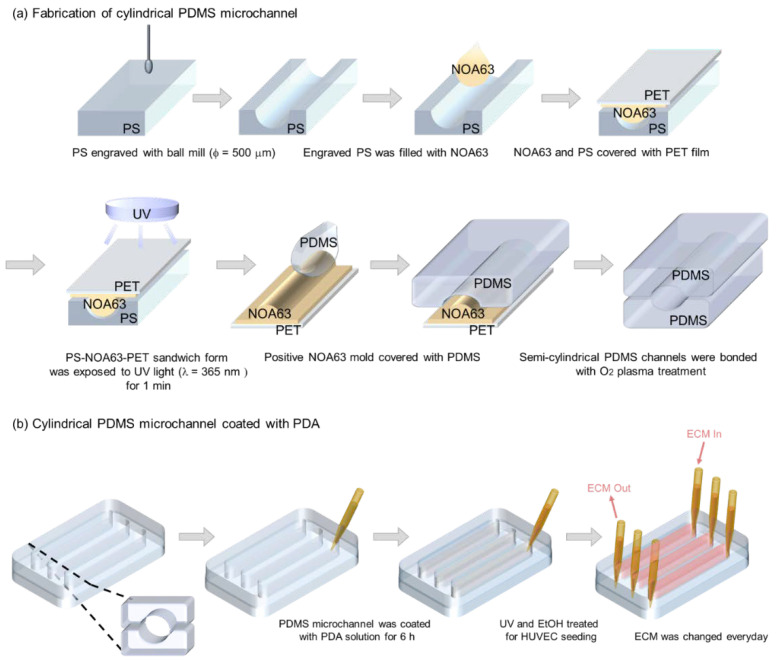
Schematic illustrations showing (**a**) the fabrication process of a cylindrical PDMS microchannel and (**b**) the PDA coating process followed by HUVEC culture.

The cylindrical PDMS microchannel was fabricated using the non-photolithographic plastic-mold-based microchannel fabrication process described in our previous studies [28,29]; the schematic is illustrated in Figure 2. Briefly, the semi-cylindrical form was engraved on a PS substrate using a computer numerical control (CNC) milling machine and a ball mill with a diameter of 1 mm. The engraved PS was then covered with NOA 63, a UV-curable polymer, then covered with PET film and pressed. The polymer filled the concave regions of the PS and was cured under UV light (365 nm, 15 mW/cm^2^) for 2 min and then the positive NOA63 mold was peeled off the PS and was affixed to a petri dish. To obtain a semi-cylindrical PDMS mold, the PDMS pre-polymer and curing agent were mixed at the same ratio as mentioned above and poured onto the petri dish, before the PDMS mixture was cured. The two semi-cylindrical PDMS molds were treated with O_2_ plasma (Femto Science, Hwaseong-si, Gyeonggi-do, Korea) for 1 min at 60 W and then bonded at 80 °C for 30 min, assisted by ethanol treatment. After the assembly, the cylindrical PDMS microchannel measuring 1 mm in diameter was coated with the PDA solution, as described above. A rectangular microchannel with a 1 × 1 mm square cross-section was used for comparison.

## 3. Results and Discussion

### 3.1. Characterization of the PDA-Coated PDMS Surface

#### 3.1.1. Water Contact Angle Measurement

**Figure 3:**
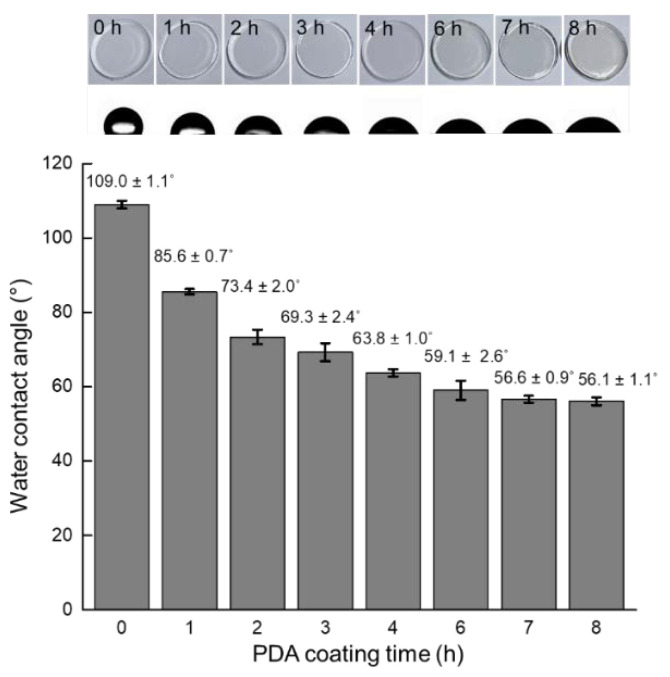
Water contact angles measured on the PDA-coated PDMS surface after coating for 0, 1, 2, 3, 4, 6, 7, and 8 h.

Figure 3 shows the water contact angle measurement conducted on the PDMS surface as a function of the PDA coating time. When PDA is polymerized at RT under alkaline conditions, it has a brownish color that gets darker with the lapse of coating time. As shown in Figure 3, the color of the PDMS surface did not change noticeably and maintained its innate transparency for up to 4 h of coating. However, when coated for 6 h, a brownish color became apparent. The uncoated bare PDMS surface had a water contact angle of 109.0 ± 1.1°, which means that the surface was hydrophobic. However, as the PDA coating time increased to 1, 2, 3, and 4 h, the water contact angle steadily decreased to 85.6 ± 0.7°, 73.4 ± 2.0°, 69.3 ± 2.4°, and 63.8 ± 1.0°, respectively. These results proved that the hydrophobic nature of the PDMS surface was rendered hydrophilic by PDA coating, which was similarly shown in our previous study [30]. The contact angles measured on the PDMS surface after it was coated with PDA for 6, 7, and 8 h were 59.1 ± 2.6°, 56.6 ± 0.9°, and 56.1 ± 1.1°, respectively, and the differences can be considered negligible. Therefore, PDA was coated for 6 h in subsequent experiments.

#### 3.1.2. XPS Measurement

**Figure 4:**
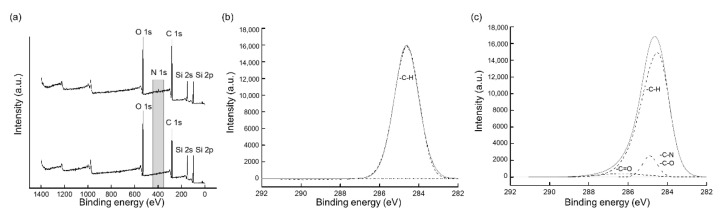
XPS analyses performed on bare and PDA-coated PDMS surfaces. (**a**) Survey spectrum, (**b**) high-resolution spectrum of bare PDMS−C1s, and (**c**) high-resolution spectrum of PDA-coated PDMS−C1s.

Figure 4a shows the results of the XPS survey spectrum for the bare PDMS surface and the surface coated with PDA for 6 h. In contrast to the former, a peak of N1s was observed in the survey spectrum of the PDA-coated PDMS surface. The atomic ratios obtained from the XPS analyses were 32.1% for O1s, 46.4% for C1s, and 21.5% for Si2p on a bare PDMS, and no N1s peak was detected. At the same time, the atomic ratios of the PDA-coated PDMS were 27.5% for O1s, 53.7% for C1s, 17.1% for Si2p, and 1.7% for N1s because dopamine contains nitrogen. Furthermore, the minor reduction observed in the Si2p ratio occurred at the same time as the N1s peak, indicating that the PDMS surface was coated with PDA. Figure 4b,c show the high-resolution C1s spectra for each surface. Only the C–H bond, with an energy of 284.6 eV, was observable on the bare PDMS; however, at least two additional peaks were detected on the PDA-coated one. A value of 288.3 eV was used for the C=O bond and a value of 286 eV was used for C–N or C–O bond deconvolution. We confirmed that chemical bonds such as C=O, C–N, and C–O were present on the PDMS surface after PDA coating, which is in line with our previous findings [31,32]. Overall, we concluded that the PDA was polymerized and the bare PDMS surface was successfully coated using the method introduced in this study.

### 3.2. Cell Culture on Bare PDMS and PDA-Coated PDMS Surfaces

**Figure 5:**
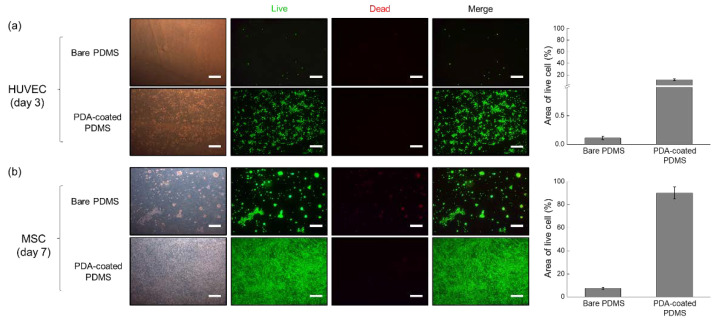
Results of the live/dead cell assays performed on HUVECs and MSCs cultured on bare and PDA-coated PDMS surfaces. The percentage of the area occupied by live cells was calculated using the ImageJ software. (**a**) HUVECs were cultured for 3 days and (**b**) MSCs were cultured for 7 days. Scale bar = 500 μm.

The results of the cytotoxicity assay after cells were cultured on bare PDMS or PDA-coated PDMS surfaces are shown in Figure 5. The image shown in Figure 5a was taken 3 days after the HUVECs were seeded; the cells attached poorly to the bare PDMS surface, and their proliferation and viability were also constrained. This is in line with observations made in previous research, demonstrating that HUVECs did not adhere to the bare PDMS surface in the absence of treatment [33]. On the other hand, when HUVECs were seeded onto the PDA-coated PDMS surface, they spread out evenly, attached, and proliferated. The cytotoxicity assay results suggest that the cytotoxicity of this surface to HUVECs was also low, further indicating that the PDA-coated PDMS surface can provide a favorable environment for the growth of HUVECs compared to the bare PDMS one. The effect of the PDA-coated PDMS on MSCs is shown in Figure 5b. On the bare PDMS surface, the cells formed aggregates with an irregular distribution, which was believed to be due to the weak cell-surface adhesion force [34,35]. Cells did not adhere to the surface due to a relatively weak interaction between the cell and the surface, and cell aggregates formed as a result of the contractile force of the cells [35]. It could be seen that a bare PDMS surface hindered the adhesion and growth of MSC, as observed by the red fluorescence, which represents dead cells stained by EthD-1 in the cell viability assay. The PDA-coated PDMS surface, on the other hand, effectively promoted cell proliferation, which means that MSCs were well attached, multiplied, and had a high viability compared to cells cultured on the bare PDMS. Ding et al. demonstrated that PDA coating promoted cell attachment and growth by upregulating the adsorption of cell-adhesive proteins [36]. Similarly, Nie et al. reported that PDA-coated surfaces enhanced human-induced pluripotent stem cell (hiPSC) adhesion and proliferation while still retaining their pluripotency [37]. Collectively, our results also ascertained that the PDA-coated PDMS surface had a positive effect on both types of tested cells.

### 3.3. MSC Differentiation on the PDA-Coated PDMS Surface

**Figure 6:**
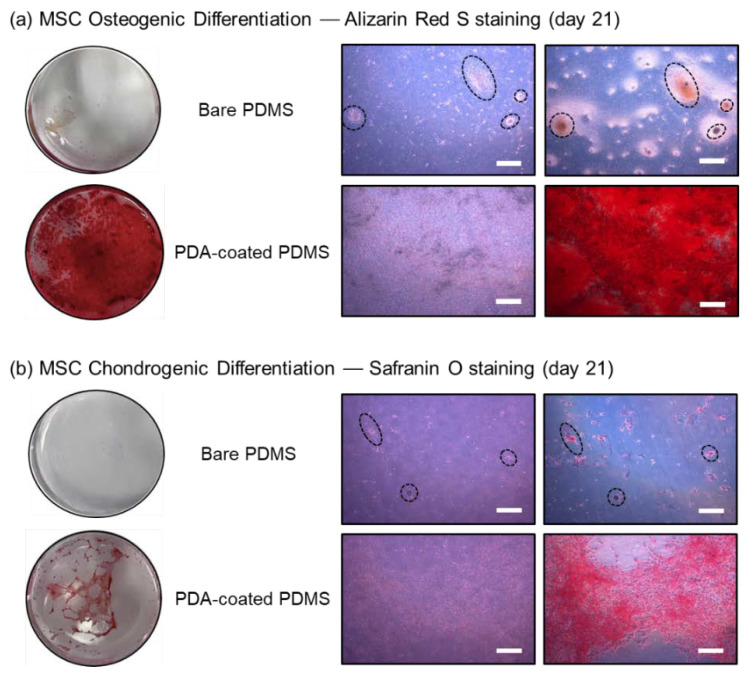
Staining results showing MSC differentiation after 21 days of culture. (**a**) Osteogenic and (**b**) chondrogenic differentiation was evaluated by staining with Alizarin Red S and Safranin O, respectively. The dotted circles and ellipses represent the same spot in the images. Scale bar = 500 μm.

Figure 6 shows the results of MSC differentiation. When MSC confluency reached 100%, MODM or MCDM were used to initiate differentiation into osteocytes or chondrocytes, respectively, and the cells were cultured for two additional weeks before staining was performed to assess the degree of differentiation. Alizarin Red S, which stains minerals generated by osteocytes, such as Ca^2+^, was used to evaluate the osteogenic differentiation of MSCs. As shown in Figure 6a, the dark red color indicated that more minerals were secreted, demonstrating that MSCs successfully differentiated into osteocytes. Since the cells were not uniformly distributed on the bare PDMS surface and therefore robust cell adhesion or proliferation was not achieved, the level of differentiation into osteocytes indicated by the staining was sparse. These results are in line with those reported in previous studies [23,38], demonstrating that PDA has a positive effect on the enhancement of MSC osteogenesis.

Chondrogenesis was evaluated via staining with Safranin O, and red-stained cells indicated that chondrogenic differentiation was well induced, as shown in Figure 6b. Alkaline Safranin O is used to verify the differentiation of chondrocytes, as it mainly stains acidic proteoglycans, which are typically found in chondrocytes. On the bare PDMS surface, it was difficult to observe chondrogenic differentiation, and only the clustering of tiny, red dot-shaped cells was visible, as observed in the osteogenic staining results. Meanwhile, chondrogenic cells induced on the PDA-coated PDMS surface aggregated over time and the transparent PDMS surface gradually began to reappear during this process. Nearly half of the PDA-coated PDMS surface that had been previously entirely covered with the MSCs during initial differentiation had recovered to the original PDMS surface by day 21, which was the last day of the induced differentiation, and the cells were clustered rather than forming a monolayer. This is in line with observations made in our previous studies, where chondrogenesis was induced under 2D conditions; cells with a cobblestone-like shape were observed; and the condensation of MSCs had progressed remarkably, leading to the formation of cell aggregations [39,40]. As a result, we believe that the PDA-coated PDMS surface can successfully induce both the osteogenic and chondrogenic differentiation of MSCs and that PDA coating can alter the surface wettability of PDMS favorable for stem cell culture and differentiation.

### 3.4. HUVEC Culture on the PDA-Coated Cylindrical PDMS Microchannel Mimicking Human Blood Vessel

**Figure 7:**
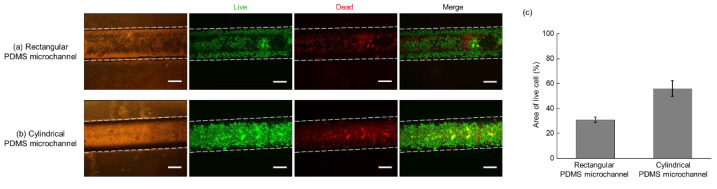
Results of the live/dead cell assay performed on HUVECs cultured in a PDA-coated PDMS microchannel for 3 days. (**a**) Rectangular, (**b**) cylindrical PDMS microchannels, and (**c**) the percentage of area occupied by live cells in each channel. Scale bar = 500 μm.

The result of HUVECs culturing on PDA-coated PDMS microchannels is shown in Figure 7. Figure 7a,b show microscopic images of the live/dead cell assays performed inside the 1 × 1 mm rectangular and cylindrical microchannel with a radius of 1 mm, respectively. Generally, cylindrical microchannels are selected as general topography for mimicking blood vessels in the body because they allow for the homogeneous spreading of cells and are regarded as suitable models for considering fluidic flow such as blood flow [41]. In this study, HUVECs cultured in the cylindrical microchannel adhered relatively homogeneously to its surface, successfully forming a monolayer of cells. On the contrary, HUVECs cultured in the rectangular PDMS, the entire surface of which was coated with PDA, were dead in a relatively high percentage as compared to those cultured inside a cylindrical microchannel, reflected by cell staining with EthD-1. In particular, cells did not seem to attach well in the central region of the microchannel, and the cell distribution was irregular. It is believed that the right-angle edge of the rectangular microchannel was a topographical hindrance to the cell and medium flow, implying that it interfered with the successful culture of the HUVECs. These results further indicate that the cylindrical PDMS microchannel coated with PDA is a more suitable platform than the rectangular one for the simulation of blood vessels in vitro.

## 4. Conclusions

In this study, we used PDA to facilitate the adhesion of cells to a PDMS elastomeric surface, which poses many advantages for cell culture, although the surface is hydrophobic, which hinders cell attachment. To evaluate the feasibility and versatility of the use of a PDA-coated PDMS surface as a cell-friendly culture platform, cells with different properties, i.e., HUVECs and MSCs, were cultured. The performance as a cell culture platform was verified by the successful differentiation of MSCs into chondrocytes and osteocytes, assisted by the application of suitable differentiation media. Furthermore, by fabricating a cylindrical microchannel and culturing HUVECs in it, this study demonstrated that the platform can be used to mimic a human blood vessel. Unlike the bare PDMS surface, the PDA-coated PDMS surface was cell-friendly, allowing for the in vivo simulation of 3D microenvironments of the human body. These results may contribute to the understanding of the design principle to properly manipulate microenvironments in vivo in a simple, cost-effective, and mass-producible manner for the study of complicated cell behaviors in vitro.
